# Indications and Outcomes of Endoscopic Gastric Pouch Plications After Bariatric Surgery: An Analysis of the Metabolic and Bariatric Surgery Accreditation and Quality Improvement Program (MBSAQIP) Database

**DOI:** 10.1007/s11695-025-07697-9

**Published:** 2025-02-18

**Authors:** Mélissa V. Wills, Juan S. Barajas-Gamboa, Gustavo Romero-Velez, Andrew Strong, Salvador Navarrete, Ricard Corcelles, Carlos Abril, Juan Pablo Pantoja, Alfredo D. Guerron, John Rodriguez, Matthew Kroh, Jerry Dang

**Affiliations:** 1 Cleveland Clinic Lerner College of Medicine, Cleveland Clinic Foundation, Case Western Reserve University, 9500 Euclid Avenue, Cleveland, OH 44195 USA; 2 Cleveland Clinic Abu Dhabi, Abu Dhabi, United Arab Emirates

**Keywords:** Bariatric surgery, Endoscopic gastric pouch plication, Revisional bariatric surgery, Metabolic and Bariatric Surgery Accreditation and Quality Improvement Program (MBSAQIP), Advanced endoscopy

## Abstract

**Background:**

Endoscopic gastric pouch plications (EGPP) have emerged as a novel approach for managing weight-related issues and postoperative complications following bariatric surgery. However, safety for these revisions remains limited. This study aims to evaluate the 30-day rate of serious complications and mortality associated with EGPP using the MBSAQIP database.

**Methods:**

A retrospective analysis of the MBSAQIP database from 2020 to 2022 was conducted, focusing on patients undergoing EGPP. The primary outcomes were 30-day serious complications and mortality.

**Results:**

The study included 1474 patients. Recurrent weight gain was the most common indication for EGPP (71.9%), followed by suboptimal initial weight loss (15.1%), dumping syndrome (5.5%), reflux (4.1%), gastrointestinal tract fistula (1.0%), and others (0.9%). The mean operative time was 41.2 ± 35.2 min, with a mean hospital stay of 0.4 ± 0.7 days. Postoperative complications included 30-day readmissions (3.1%), serious complications (3.3%), 30-day interventions (2.5%), bleeding (0.8%), and reoperations (0.4%). The mortality rate was 0%. Multivariable analysis identified GERD as an independent predictor of serious complications (OR 1.79, 95% CI 0.98 to 3.2, *p* = 0.05) when adjusting for various factors.

**Conclusions:**

EGPP is an uncommon procedure with only 1474 cases reported, primarily indicated for weight recurrence. It appears to be a relatively safe alternative to surgical revision. However, further research is needed to assess its efficacy and compare it to corresponding surgical revisions.

## Introduction

Bariatric surgery has emerged as a pivotal intervention in the management of obesity, offering sustainable weight loss for patients who have not achieved success with non-operative methods. The Roux-en-Y gastric bypass (RYGB) stands out as the gold standard procedure, consistently achieving approximately 30% total body weight loss within the first 12 months post-surgery [1]. In the USA, around 300,000 bariatric surgeries are performed annually, with RYGB accounting for nearly a third of these operations and demonstrating remarkable efficacy in improving obesity-related comorbidities [2].

However, like other operations, RYGB is vulnerable to complications that can require re-intervention and re-operation. It has been estimated that excessive weight recurrence occurs in 37% of patients at 1 year after surgery [3]. The causes are multifactorial and include dilation of gastrojejunal anastomosis (GJA), elongation of the gastric pouch, and less commonly, gastro-gastric fistulas [4]. Other complications that necessitate revisional surgery include leaks, fistulas, dumping syndrome, or gastroesophageal reflux disease (GERD). It is estimated that 12% of patients will have some type of surgical revision following gastric bypass surgery, either for unsatisfactory weight loss or for complications [5]. Surgical options to address these complications include revision of the GJA or pouch, placement of adjustable gastric bands, and distalization of the gastric bypass [6]. However, traditional revisional surgery is associated with significant operative risks. As overall access to bariatric surgery increases and as the complexity of patients who are offered these operations broadens, the pool of patients that would benefit from a lower risk revisional intervention also increases. To this end, minimally invasive revisional procedures including endoscopic gastric pouch plication (EGPP), in which endoscopic suturing is used to tighten the dilated gastric pouch [7], have been developed. In contrast to traditional RYGB revisions, EGPP may incur fewer risks typically associated with laparoscopic or open surgery, such as long operating room times and post-operative pain.

Endoscopic procedures generally have lower complication rates than traditional surgical revisions, but current knowledge about endoscopic revisions of bariatric surgery is limited, primarily based on case reports, single-center experiences, and literature reviews. To date, no large-scale study has analyzed the safety outcomes of EGPP or identified risk factors for complications after this procedure. This study advances our understanding by utilizing the Metabolic and Bariatric Surgery Accreditation and Quality Improvement Program (MBSAQIP) database, leveraging its large-scale, standardized data from accredited US centers to produce the first comprehensive analysis of EGPP safety outcomes and predictors of complications.

## Materials and Methods

### Study Design and Ethical Approvals

A retrospective analysis of the MBSAQIP data registry was performed. Only the years 2020 to 2022 were included because the registry was modified in 2020 to include important details on revisional procedures. The MBSAQIP currently captures clinical data from 902 accredited American and Canadian centers. The data registry prospectively collects data and contains standardized pre-, intra-, and post-operative variables specific to bariatric surgery patients. This study was reviewed and deemed exempt by the Institutional Review Board (IRB) due to the anonymity of the data.

### Objectives

The primary objectives of this study were to determine the rate of serious complications and mortality of endoscopic gastric pouch plication in the revisional setting, and to identify independent predictors of serious complications through multivariable analysis. Patients with at least one of the following complications within 30 days of surgery were defined as having a serious complication: anastomotic leak, postoperative bleeding, reoperation, non-operative intervention, readmission, any cardiac event (cardiac arrest, MI, or cardiopulmonary resuscitation), pneumonia, acute kidney injury, venous thromboembolic event, sepsis, unplanned intubation, cerebrovascular event, other serious complications, or death.

### Inclusion and Exclusion Criteria

Patients were identified if they were undergoing a revisional procedure (variable REV-PROC) labelled in the MBSAQIP participant use file as “Gastric pouch or stoma plication or revision.” Surgical gastrojejunal revisions were excluded by only including “Endoscopic” surgical approaches.

### Patient Variables

Basic demographic data including age, sex, race, and body mass index (BMI) were collected. Patient comorbidities included the following: tobacco use, diabetes (categorized as non-diabetic or diet-controlled, insulin-dependent, and non-insulin dependent), hypertension, gastroesophageal reflux disease (GERD), chronic obstructive pulmonary disease, hyperlipidemia, chronic steroid use, renal insufficiency, preoperative dialysis, history of venous thromboembolism, of deep venous thrombosis, of pulmonary embolism, and of venous stasis, therapeutic anticoagulation use, sleep apnea, history of myocardial infarction (MI), and history of percutaneous coronary intervention. Functional status variables encompassed preoperative functional status (defined as independent, partially dependent, or fully dependent) and American Society of Anesthesiologists (ASA) Physical Status classification.

The identification process utilized a comprehensive review of diagnostic codes associated with each of these events. Specifically, the analysis examined up to 4 readmission diagnoses, 9 intervention diagnoses, and 14 reoperation diagnoses for each patient. Additionally, the cause of death was scrutinized when applicable.

Ulcers and strictures were identified as the cause of readmission, non-operative reintervention, reoperation, or death. For each of these clinical events, the presence of specific diagnostic terms—“Anastomotic ulcer,” “Gastric ulcer,” “Gastrointestinal tract ulcer without perforation” for ulcers, and “Gastrointestinal tract stricture or obstruction” for strictures—was used to flag the respective complication.

### Technical Procedure

**Fig. 1:**
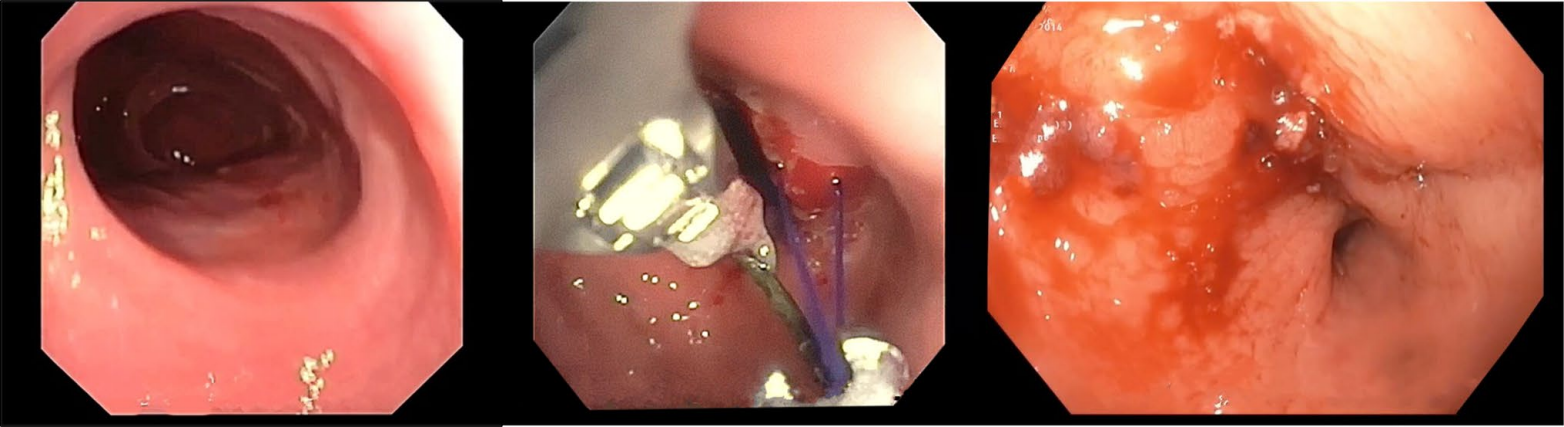
Endoscopic revision of dilated gastrojejunal anastomosis. **Left:** initial endoscopic view demonstrating a dilated gastrojejunal (GJ) anastomosis measuring > 25 mm in diameter in a 47-year-old female with weight recurrence following Roux-en-Y gastric bypass. **Center:** intraoperative endoscopic view demonstrates the placement of an endoscopic suturing system. A helical tissue grasper is used to facilitate full-thickness tissue acquisition while placing interrupted permanent sutures from the jejunal to the gastric side of the anastomosis. **Right:** final endoscopic view reveals the successfully revised GJ anastomosis with a patent but significantly reduced aperture after placement of three interrupted sutures and T-tag anchoring

EGPP is an advanced endoscopic procedure that requires specialized training in bariatric endoscopy. The procedure involves endoscopic visualization of the gastric pouch and gastrojejunal anastomosis, followed by placement of full-thickness or partial-thickness endoscopic sutures using devices such as the Apollo OverStitch or Incisionless Operating Platform (Fig. 1). These sutures are placed in a continuous or interrupted fashion to reduce the pouch volume and/or outlet diameter. The procedure typically requires general anesthesia and CO2 insufflation. Practitioners performing EGPP should have expertise in therapeutic endoscopy and typically complete dedicated training programs in bariatric endoscopy, with specific emphasis on endoscopic suturing techniques.

### Statistical Analysis

Categorical data were expressed as percentages, and continuous data as weighted mean and standard deviation (SD). A multivariable logistic regression analysis was used to identify predictive factors for serious complication and mortality within 30 days. The available case method addressed missing data as all variables had less than 5% missingness. Patient factors and operative time were included in the model. Any variable with a *p*-value < 0.05 in univariate analysis was included in multivariable analysis. Variables were checked for collinearity via the variable inflation factors method. All statistical analyses were completed using STATA 17 statistical software (StataCorp, College Station, TX, USA).

## Results

### Patient Demographics

The study population had a mean age of 50.6 years (SD ± 9.9). The majority of patients were female (89.9%). Racially, the cohort was predominantly white (60.3%), followed by black (26.8%), and other races (12.8%). Patients had an initial mean BMI of 40.4 kg/m^2^ (SD ± 7.9). Most patients were classified as ASA 3 (61.8%), indicating moderate systemic disease. Comorbidities were common, with hypertension (36.7%), gastroesophageal reflux disease (34.6%), and sleep apnea (19.6%) being the most prevalent. Diabetes was present in 13.2% of patients, with 10.5% being insulin-dependent (Table 1).

**Table 1:** Demographics, baseline characteristics, and significant risk factors for serious complications on multivariable logistic regression

Characteristic	Count (%) or mean (± SD)	Multivariable logistic regression		
		Adjusted odds ratio	95% confidence interval	*p-*value
Age (years	50.6 ± 9.9	1.02	0.99–1.05	0.11
Female	1326 (89.9)			
Race				
White	890 (60.3)			
Black	395 (26.8)			
Other	189 (12.8)			
Mean body mass index (kg/m^2^) ± SD	40.4 ± 7.9			
ASA class				
1–2	521 (35.4)			
3	910 (61.8)			
4–5	40 (2.7)			
Tobacco use	48 (3.2)			
Diabetes				
Non-diabetic or diet-controlled	1277 (86.6)			
Non-insulin dependent	41 (2.7)	1.83	0.54–6.20	0.33
Insulin dependent	156 (10.5)	0.86	0.33–2.25	0.77
Hypertension	135 (36.7)			
Gastroesophageal reflux disease	510 (34.6)	1.79	0.99–3.25	*0.05
Chronic obstructive pulmonary disease	6 (0.4)			
Hyperlipidemia	224 (15.2)	1.42	0.66–3.01	0.37
Chronic steroid use	10 (2.7)			
Chronic kidney disease	3 (0.2)	3.90	0.27–55.5	0.32
Dialysis dependent	5 (0.3)			
History of venous thromboembolism	62 (4.2)	2.28	0.84–6.23	0.11
History of deep venous thrombosis	42 (2.9)			
History of pulmonary embolism	32 (2.2)			
History of venous stasis	9 (0.6)			
Preoperative therapeutic anticoagulation	47 (3.2)	2.59	0.92–7.28	0.07
Sleep apnea	289 (19.6)	1.32	0.68–2.57	0.40
History of myocardial infarction	13 (0.9)			
History of previous cardiac surgery	10 (0.7)			
History of percutaneous coronary intervention	18 (1.2)			

*SD* Standard deviation, *ASA* American Society of Anesthesiologists

^*^p < 0.05

### Indications for Revision

The primary indications for EGPP were predominantly weight-related issues. Recurrent weight gain was the most common reason, accounting for 71.9% of cases, followed by suboptimal initial weight loss at 15.1%. Gastrointestinal complications were also significant indications, with dumping syndrome (5.5%) and GERD (4.14%) being the most prevalent. Less common indications included gastrointestinal tract fistula (1.0%), persistent comorbidities (0.5%), and hypoglycemia (0.3%). Rare indications comprised abdominal pain, anastomotic or staple line leak, nausea and/or vomiting, dysphagia, and patient intolerance, each accounting for less than 0.5% of cases. Other unlisted reasons made up 1.0% of the indications (Table 2).

**Table 2:** Indications for revision

Indication	Count (%)
Recurrent weight gain	1060 (71.9)
Inadequate weight loss	223 (15.1)
Dumping syndrome	81 (5.5)
Gastroesophageal reflux disease	61 (4.1)
Gastrointestinal tract fistula	15 (1.0)
Persistent comorbidities	7 (0.5)
Hypoglycemia	5 (0.3)
Abdominal pain	3 (0.2)
Anastomotic or staple line leak	2 (0.1)
Nausea and/or vomiting	1 (0.1)
Dysphasia	1 (0.1)
Patient intolerance	1 (0.1)
Other (not listed)	14 (1.0)

### Procedural Factors, Complications, and Mortality

The average operating time for EGPP was 41.2 min (SD 35.2). The mean hospital stay length was 0.4 days (SD 0.7) with 72.1% (*n* = 1063) of cases performed as a same-day procedure. The rates of complications for patients undergoing EGPP are as follows: anastomotic leak (0.3%), postoperative bleed (0.9%), reoperation (0.4%), re-intervention (2.6%), readmission (3.2%), cardiac complication (0.07%), pneumonia (0.07%), acute kidney injury (0.07%), venous thromboembolism (0.07%), deep surgical site infection (0.3%), and serious complications (3.3%). No patients experienced sepsis, unplanned intubation, or cerebrovascular accident. There were no mortalities recorded in the cohort (Table 3).

**Table 3:** 30-day postoperative complications

Complication	Count (%)
Anastomotic leak	4 (0.3)
Any bleed	13 (0.9)
Reoperation	6 (0.4)
Intervention	38 (2.6)
Readmission	47 (3.2)
Any cardiac event	1 (0.07)
Pneumonia	1 (0.07)
Acute kidney injury	1 (0.07)
Venous thromboembolism	1 (0.07)
Deep surgical site infection	4 (0.3)
Wound disruption	0 (0.0)
Sepsis	0 (0.0)
Unplanned intubation	0 (0.0)
Cerebrovascular accident	0 (0.0)
Ulcers	7 (0.5)
Strictures	2 (0.1)
Serious complications	49 (3.3)

### Multivariable Logistic Regression Analysis

Following adjustments with multivariable logistic regression, only GERD was independently predictive of serious complications (OR 1.79, 95%, CI 0.99 to 3.25, *p* = 0.05). Notably patients with GERD had more than double the rate of serious complications (5.1 vs 2.4%, p = 0.006). No multivariable mortality analysis was conducted as there were no deaths (Table 1).

### Subgroup Analysis for GERD Versus Non-GERD

Most complications occurred at similar rates in both groups, with no statistically significant differences for leaks, bleeding, interventions within 30 days, cardiac events, acute kidney injury, venous thromboembolism, and surgical site infections. However, significant differences were observed in reoperation rates (1.0% vs 0.01%, *p* = 0.012), readmission within 30 days (2.4% vs 4.7%, *p* = 0.016), and ulcer occurrence (0.2% vs 1.0%, *p* = 0.040), with GERD patients experiencing higher rates in these categories. The group with GERD also experienced more frequent strictures (0.0% vs 0.2%). Notably, the overall rate of serious complications was significantly higher in the GERD group (2.4% vs 5.1%, *p* = 0.006). Some complications, such a sepsis, unplanned intubation, and CVA, did not occur in either group. This analysis suggests that patients with pre-existing GERD may be at higher risk for certain postoperative complications following this procedure. (Table 4).

**Table 4:** Subgroup analysis for GERD versus Non-GERD

	Number of patients with complications		
30-day complication	No GERD (*n* = 964, %)	GERD (*n* = 510, %)	*p*-value
Leak	2 (0.2%)	2 (0.4%)	0.517
Bleed	7 (0.7%)	6 (1.2%)	0.379
Reoperation	1 (0.1%)	5 (1.0%)	*0.012
Non-operative intervention	20 (2.1%)	18 (3.5%)	0.094
Readmission	23 (2.4%)	24 (4.7%)	*0.016
Cardiac event	0 (0.0%)	1 (0.2%)	0.169
Pneumonia	0 (0.0%)	1 (0.2%)	0.169
Acute kidney injury	1 (0.1%)	0 (0.0%)	–
Venous thromboembolism	0 (0.0%)	1 (0.2%)	0.169
Deep surgical site infection	2 (0.2%)	2 (0.4%)	0.517
Wound disruption	0 (0.0%)	0 (0.0%)	–
Sepsis	0 (0.0%)	0 (0.0%)	–
Unplanned intubation	0 (0.0%)	0 (0.0%)	–
Cerebrovascular accident	0 (0.0%)	0 (0.0%)	–
Serious complication	23 (2.4%)	26 (5.1%)	*0.006
Ulcers	2 (0.2%)	5 (1.0%)	*0.040
Strictures	0 (0.0%)	2 (0.2%)	–

^*^p < 0.05

## Discussion

To the best of our knowledge, this is the first large-scale study analyzing the rate of serious complications and mortality of EGPP after RYGB for recurrent weight gain, suboptimal initial weight loss, dumping syndrome, and GERD. Using the MBSAQIP database, we found the overall rate of complications after EGPP to be relatively low with a mortality of 0%. We also found GERD to be an independent predictive factor of serious complications.

The association between GERD and serious complications is unclear and may be multifactorial. Iatrogenic trauma from the endoscopic procedure and friable gastric tissue seen in reflux and gastritis could contribute [8, 9], although we did not find a significantly higher bleeding rate in this group. It may also reflect that patients with GERD may have had prior endoscopic procedures, thus placing them at higher risk of complications with repeated endoscopy used for EGPP [10]. Finally, this could be due to the greater risk of ulceration in patients with GERD due to the higher acidity in the gastric pouch. Indeed, though RYGB is considered superior to other bariatric surgeries in terms of symptomatic control of GERD, emerging studies have also shown new onset GERD after RYGB [11]. The presence of GERD in this cohort may suggest larger pouch sizes, which have been associated with higher rates of ulcers, potentially due to the persistence of parietal cells in oversized gastric pouches. This could reduce the effectiveness and safety of gastric plication [12, 13]. Marginal ulcers are known risks of bariatric surgery and can lead to severe complications—one study demonstrated a 1.8% incidence of melena secondary to marginal ulceration [14]. It is worth noting that the variable *GERD* included in the MBSAQIP registry refers to the existence of a pre-operative GERD diagnosis. The methods for assigning these diagnoses lack standardization, making it difficult to ascertain specific details about the diagnostic process. It may include intermittent heartburn symptoms, reduction in symptoms with anti-secretory medications, endoscopic evidence of esophagitis or intestinal metaplasia, radiographic studies demonstrating retrograde flow of contrast, impedance studies showing acid or non-acid reflux among others. Additionally, the database does not capture information about associated hiatal hernias or their repairs, which could be important confounding factors in the relationship between GERD and post-EGPP complications. Gastric bypass reflux may be acidic or non-acidic in nature, an important distinction as these different mechanisms may impact symptom patterns, treatment outcomes, and operative compliactions. More research is needed to investigate the risks and benefits of EGPP in patients with GERD, with particular attention to clearly defined criteria for inclusion.

In our analysis, recurrent weight gain and suboptimal initial weight loss were the primary indications for EGPP. Previous studies have demonstrated EGPP to be effective for treating these. Schroder et al. found that within six months, in patients with significant weight recurrence after RYGB, EGPP led to an average loss of 32% of the weight that had been regained [14]. Jirapinyo and Thompson found that in patients with suboptimal initial weight loss or weight recurrence, EGPP led to 9.5% ± 8.5% total weight loss after 12 months, and 12.5% after 5 years [15]. These studies, as well as others in the literature, demonstrate that EGPP is successful in treating weight recurrence in the long term.

Dumping syndrome (DS) also accounted for a proportion of patients undergoing EGPP (5.5%). DS consists of a cluster of symptoms including nausea, vomiting, and loose stools, induced by the rapid transit of undigested food into the small bowel. Its clinical manifestations are graded by the Sigstad’s score—a score of > 7 suggesting a diagnosis of DS [16]. Dilation of the gastro-jejunal anastomosis (GJA) has been related to DS, along with weight recurrence, after RYGB [17], and therefore, EGPP with narrowing of the GJA is a strategy for symptom mitigation. Tsai et al. reported EGPP to be effective in significantly improving the Sigstad’s score from 13.9 to 8.6 after three months [18]. Pontecorvi et al. reported EGPP to be effective in significantly decreasing symptoms of DS from 15 to 2 by 24 months [17]. It is clear that EGPP is effective for treatment of DS both within the short and long term.

Our study found that EGPP was safe regardless of indication, with a low proportion of adverse events, and no mortalities. This is largely congruent with other studies, which report similar rates of our described complications [7, 14, 19]. Others found similarly low rates of bleeding requiring blood transfusion [20]. Low rates of GJA anastomotic stenosis were also reported in the literature, though our MBSAQIP did not evaluate this specifically. These were typically treated with balloon dilation or lumen-apposing metal stent placement [21–23].

There are certain weaknesses to the present study. One of these is the absence of a comparison group. However, outcomes of revisional bariatric surgery have been extensively described in the literature, and it is therefore reasonable to cite those outcomes in the absence of a comparative cohort. This dataset only included variables pre-defined by the MBSAQIP and does not include information such as the timeframe between the index bariatric surgery and the revisional EGPP and how suboptimal initial weight loss or recurrent weight gain were defined. The MBSAQIP search also did not distinguish between the various strategies for EGPP, including the use of argon plasma coagulation and the types of plication devices and techniques, which have been demonstrated in the literature to have varying effects on weight loss and dumping syndrome. Furthermore, we do not have information regarding the factors leading to weight recurrence and suboptimal initial weight loss, which may have incurred selection bias. However, the large sample of cases examined (*n* = 1474) is helpful in mitigating this weakness and increasing the power of the study. Finally, patients were not excluded based on their procedural or surgical history—it is possible that the complications were found in patients who had a history of repeated endoscopic dilations or other procedures that may have affected the safety or success of EGPP.

Based on our analysis, we propose several key considerations for patient selection and management of EGPP candidates. The procedure appears most appropriate for patients with weight recurrence or suboptimal initial weight loss who are poor surgical revision candidates. However, careful consideration should be given before performing EGPP in patients with GERD, given their doubled risk of complications and readmissions. The procedure is well-suited for the outpatient setting, as evidenced by the 72% same-day discharge rate. Patients should be counseled about the overall risk of serious complications (3.3%), with higher rates (5.1%) in those with GERD. For GERD patients specifically, closer post-procedure monitoring may be warranted.

Future research should focus on several key areas: long-term efficacy data comparing EGPP to surgical revision, standardized criteria for patient selection (particularly regarding GERD status), cost-effectiveness analyses, and quality of life outcomes. Additionally, studies examining technical variations in EGPP techniques and their impact on outcomes would help establish best practices. Multicenter prospective trials would be particularly valuable in addressing these knowledge gaps.

## Conclusion

Our results demonstrate that EGPP is an uncommon procedure, with only 1474 cases reported in this study. Weight recurrence emerged as the predominant indication, accounting for 71.9% of all cases. EGPP presents itself as an alternative approach to surgical revision, showing a relatively favorable safety profile. However, it is crucial to note that further research is necessary to thoroughly assess its efficacy and draw comparisons with corresponding surgical revision techniques. This additional data will be essential in determining the optimal role of EGPP in the management of post-bariatric surgery complications and weight-related issues.

## Data Availability

No datasets were generated or analysed during the current study.
